# An investigation of the YidC-mediated membrane insertion of Pf3 coat protein using molecular dynamics simulations

**DOI:** 10.3389/fmolb.2022.954262

**Published:** 2022-08-15

**Authors:** Adithya Polasa, Jeevapani Hettige, Kalyan Immadisetty, Mahmoud Moradi

**Affiliations:** Department of Chemistry and Biochemistry, University of Arkansas, Fayetteville, AR, United States

**Keywords:** YidC, docking, molecular dynamics simulations, Pf3 coat protein, membrane insertion, steered molecular dynamics

## Abstract

YidC is a membrane protein that facilitates the insertion of newly synthesized proteins into lipid membranes. Through YidC, proteins are inserted into the lipid bilayer via the SecYEG-dependent complex. Additionally, YidC functions as a chaperone in protein folding processes. Several studies have provided evidence of its independent insertion mechanism. However, the mechanistic details of the YidC SecY-independent protein insertion mechanism remain elusive at the molecular level. This study elucidates the insertion mechanism of YidC at an atomic level through a combination of equilibrium and non-equilibrium molecular dynamics (MD) simulations. Different docking models of YidC-Pf3 in the lipid bilayer were built in this study to better understand the insertion mechanism. To conduct a complete investigation of the conformational difference between the two docking models developed, we used classical molecular dynamics simulations supplemented with a non-equilibrium technique. Our findings indicate that the YidC transmembrane (TM) groove is essential for this high-affinity interaction and that the hydrophilic nature of the YidC groove plays an important role in protein transport across the cytoplasmic membrane bilayer to the periplasmic side. At different stages of the insertion process, conformational changes in YidC’s TM domain and membrane core have a mechanistic effect on the Pf3 coat protein. Furthermore, during the insertion phase, the hydration and dehydration of the YidC’s hydrophilic groove are critical. These results demonstrate that Pf3 coat protein interactions with the membrane and YidC vary in different conformational states during the insertion process. Finally, this extensive study directly confirms that YidC functions as an independent insertase.

## 1 Introduction

Approximately 33% of all membrane proteins are inserted and embedded in the plasma membrane bilayer during co-translation (Krogh et al., 2001; Rapoport, 2007). The membrane proteins YidC/Oxa1/Alb3 work to fold incoming peptides into the membrane as efficiently as possible (Jiang et al., 2003; Dalbey and Kuhn, 2015; McDowell et al., 2021; Güngör et al., 2022; Nass et al., 2022). In an experimental study, over sixty cytoplasmic membrane proteins were found whose membrane insertion/folding is substantially hindered in the absence of YidC (Gray et al., 2011). YidC catalyzes the transmembrane insertion of newly synthesized membrane proteins in the absence of an energy supply domain, such as an ATPase (Dalbey et al., 2014), and is also involved in the insertion and placement of membrane proteins in microbes. The extent to which insertase proteins are required for inserting proteins into the membrane has been thoroughly investigated. They can be found in all kingdoms of life and are necessary for cell viability (Dalbey and Chen, 2004; Borowska et al., 2015; Kuhn and Kiefer, 2017; Chen et al., 2022). They are adaptable proteins and can function along with the SecYEG pathway to insert peptides into the membrane through the Signal Recognition Particle (SRP) mechanism. They can fold and insert proteins independently of the Sec pathway (Samuelson et al., 2000; Scotti et al., 2000; Dalbey and Chen, 2004; Facey and Kuhn, 2004; Lewis and Brady, 2015; Kiefer and Kuhn, 2018; Laskowski et al., 2021; Lewis and Hegde, 2021). This study primarily focuses on the conformational dynamics of YidC, including both local and global conformational changes involved in the insertion process of the Pf3 coat protein.

YidC completes its function either independently as a membrane insertase or as a chaperone in the SecYEG complex mechanism. In an experimental study, the deletion of YidC resulted in a conformational change of LacY during the insertion process by the SecYEG complex (Zhu et al., 2013). Hence, YidC plays a critical role in the insertion of the LacY lactose permease membrane layer protein (Nagamori et al., 2004; Kol et al., 2006; Lewis and Brady, 2015; Serdiuk et al., 2016; Spann et al., 2018). Also, YidC is involved in the incorporation of subunit II of cytochrome o oxidase in *E.Coli* (Van der Laan et al., 2003; Van Bloois et al., 2004; Yi and Dalbey, 2005). Initially, the Sec-autonomous pathway was thought to function without the contribution of an insertase. However, many studies have demonstrated that YidC is fundamental for the addition of small phage coat proteins like Pf3 and M13 in a Sec-free pathway (Dalbey and Chen, 2004; Yuan et al., 2007; Kol et al., 2008; Ernst et al., 2011; Klenner and Kuhn, 2012; Spann et al., 2018; Xin et al., 2018; Endo et al., 2022).

A few experimental studies have explored the role of YidC in various microbial organisms. The genomes of most gram-positive microscopic organisms encode two YidC proteins: YidC1 and YidC2 (Funes et al., 2011, 2009). Although YidC typically exists as a dimer or tetramer (Kohler et al., 2009) under physiological conditions, it was discovered that YidC can also function as a monomer in lipid bilayers (Kedrov et al., 2013; Kumazaki et al., 2014b; Dalbey et al., 2014; Spann et al., 2018). The protein is firmly anchored in the lipid bilayer by interfacial aromatic residues, a cytoplasmic salt-bridge group, and a periplasmic helix enhanced with aromatic residues. The aromatic residues above the R72 amino acid in YidC from Bacillus halodurans may offer a polar hydrophobic surface for the insertion of peptides into the lipid bilayer (Ito et al., 2010; Chen et al., 2017). The C–terminus of monomeric YidC cooperates with the ribosomes, and the short interhelical loops come into contact with the ribosomal proteins (Kedrov et al., 2016). YidC is believed to promote membrane insertion simply by binding nascent chains and promoting their insertion into the lipid bilayer using hydrophobic force (Dalbey et al., 2014). The hydrophilic groove inside the membrane core of the YidC will increase the rate of accepting the hydrophilic moieties of a substrate into the membrane (Wickles et al., 2014; Ito et al., 2019). The YidC hydrophilic region traverses the inner side of the membrane and is closed to the periplasmic side of the bilayer. This decreases the hydrophobicity of the membrane towards the external side of the lipid bilayer. This hypothesis of the YidC mechanism provides excellent opportunity to study the conformational dynamics of YidC. In the first step, it interacts with a hydrophilic protein region temporarily in its groove, and in the second step, this peptide is translocated to the periplasmic side (Kiefer and Kuhn, 2018).

**FIGURE 1:**
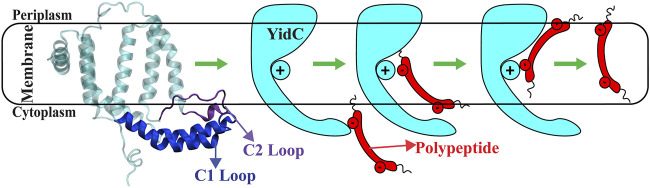
The cartoon representation of YidC and the schematic illustration of the SecY-independent insertion mechanism. A cartoon representation of YidC’s C1 and C2 loops on the cytoplasmic side (left). Schematic illustration of the YidC Sec-independent insertion of polypeptide (right).

Many prior studies have reported various explanations of the YidC independent mechanism. However, the global and local structural changes that occur in YidC during the process are not completely defined. It’s unknown how the cytoplasmic hairpin region of YidC and the water molecule inside the groove area of YidC take action during the insertion process. How would the incoming peptide or protein’s structure and conformation change during the process? We examined this topic using a combination of docking, classical molecular dynamics, and non-equilibrium simulations to analyze Sec-independent YidC’s (Figure 1) insertion of the Pf3 coat protein into the membrane. In this study, we looked at the local and global conformational changes of YidC associated with Pf3 coat protein insertion into the hydrophilic groove, Pf3 coat protein interactions with YidC and the membrane, and conformational changes in Pf3 coat protein that occurred during the insertion process.

## 2 Methods

**FIGURE 2:**
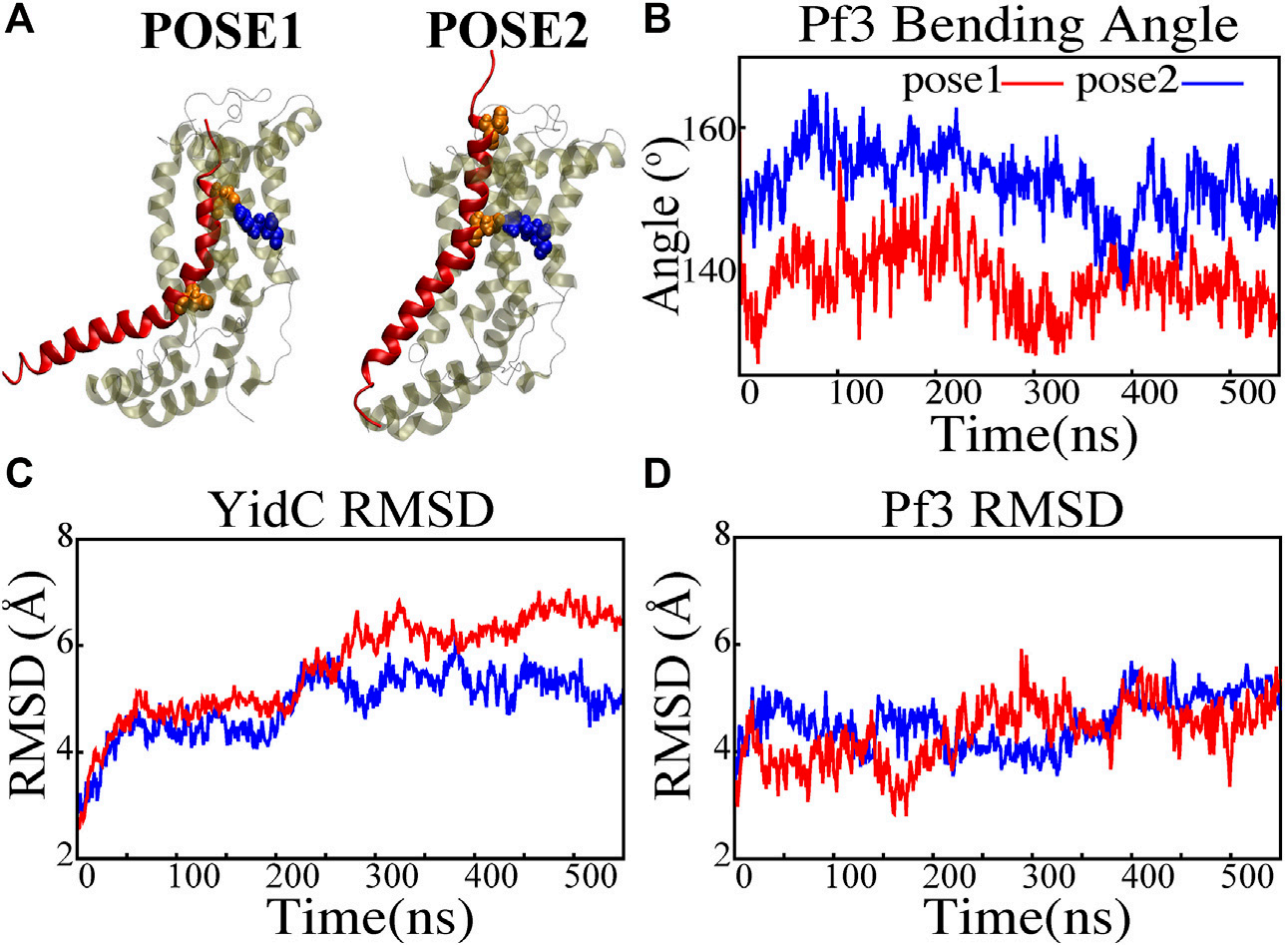
Structural stability evaluation of YidC and Pf3 coat protein in the insertion process. **(A)** Cartoon representation of pose1 and pose2 docking models generated based on the YidC (PDB:3WO7) Pf3 coat protein insertion process, as described previously in methods. **(B)** The bending angle of the Pf3 coat protein helix in pose1 (red) and pose2 (blue) models shown as a function of time. **(C,D)** Root mean square deviation of the YidC and Pf3 coat protein in pose1 (red) and pose2 (blue) models. Based on RMSD results, we have observed that in pose2 stage the YidC is significantly more stable compared to the pose1 state.

The crystal structure of YidC [PDB:3WO7 (Fujihashi et al., 2013)] was downloaded from the Protein Data Bank. Initially, the system was prepared using the Molecular Operating Environment (MOE) software [Molecular Operating Environment (MOE), 2015] by removing the water molecules from the crystal structure and assigning the appropriate protonation states for the residues using the protonate3D facility. We used MOE software to create two docking structures of the Pf3 coat protein interacting with YidC based on the previously hypothesized stages involved in the YidC Sec-independent insertion process Dalbey et al. (2014); Tsukazaki (2019); Kumazaki et al. (2014a). In pose1, Pf3 coat protein is docked in the YidC’s hydrophilic groove (Figure 2A) to evaluate probable interactions and conformational changes in the mechanism’s initial phase. The Pf3 coat protein is docked near to the periplasmic side (Figure 2A) of the protein in pose2 to identify the interactions and conformational changes involved in the mechanism’s final phase. Biased and unbiased all-atom molecular dynamics (MD) simulations were performed to characterize the conformational differences of the two bacterial YidC2 - Pf3 docking models pose1 and pose2 (Figure 2A) in a membrane environment. All simulations were performed with the NAMD 2.13 (Phillips et al., 2005) using the CHARMM36m (Huang et al., 2017) force field (Klauda et al., 2010). TIP3P (Jorgensen et al., 1983) waters were used to solvate the protein. YidC was inserted into the lipid bilayer, solvated, and ionized using the membrane builder on CHARMM-GUI (Jo et al., 2007). In these MD studies, we used palmitoyloleoyl phosphatidylethanolamine (POPE) lipids to build a lipid bilayer. A membrane layer surface of 110 Å  × 110 Å  was built along the XY plane. The protein lipid-assembly was solvated in water with 25 Å  thick layers of water on top and bottom. 0.15 M of Na^+^ and Cl^−^ ions were added to the solution with a slight modification in the number of ions to neutralize the system. The solvated system contained ≈ 142,000 atoms. Before the equilibrium simulation, the structure was energy minimized using the conjugate gradient algorithm (Reid, 1971). Following that, we used the standard CHARMM-GUI (Jo et al., 2007) protocol to progressively relax the systems using restrained MD simulations. In the NPT ensemble at 310 K, 550 ns of equilibrium MD simulations were performed under periodic boundary conditions for each system. In the simulations, a Langevin integrator with a damping coefficient of *γ* = 0.5 ps^−1^ and 1 atm pressure was maintained using the Nose-Hoover Langevin piston method (Martyna et al., 1994; Feller et al., 1995).

Trajectories were visualized and analyzed using VMD software (Humphrey et al., 1996). Salt bridge interaction analysis was conducted via VMD plugins. For salt bridge analysis, the cut-off distance was set at 4 Å and the distance between the oxygen atoms of the acidic residues and the nitrogen atoms of basic residues were calculated. The interhelical angles were calculated as the angle between the third principal axes of the corresponding helices (Immadisetty et al., 2022, 2017, 2019). The TM helices and other sub-domains were defined for analysis as follows: TM1a (58–78), TM1b (79–104), TM2 (134–155), TM3 (175–190), TM4 (219–233), TM5 (233–258), C1 region (84–133), C2 loop (195–216), and modified C-terminal region (256–272) respectively. The number of contacts within 3 Å of selection was measured for contact analysis. We counted the number of water molecules within 5 Å of R72 for water analysis. For the Pf3 coat protein bending angle, we chose two pairs of residues selection for the top (ASP7-ASP18) and bottom (ASP18-LEU29) regions of Pf3 coat protein and measured their third principal axes, denoted by *v*_1_ and *v*_2_, respectively. The angle between the two vectors was calculated as $180{}^{\circ}-\arccos\left(\frac{v_{1}\cdot v_{2}}{\left|v_{1}\|v_{2}\right|}\right)$ . Principal component analysis (PCA) was performed on each trajectory using PRODY (Bakan et al., 2011) software. Only *C*_*α*_ atoms of the peptide were considered in the PCA calculations of both docking simulations. The VMD plugin MEMBPLUGIN was used to calculate the area per lipid and the deterium order parameter of equilibrated POPE membranes (Guixà-González et al., 2014).

The combination of equilibrium and non-equilibrium MD simulations has proven effective for investigating biological challenges (Govind Kumar et al., 2022; Immadisetty et al., 2021; Polasa et al., 2022; Moradi et al., 2015, 2011, 2014a,b; Moradi and Tajkhorshid, 2013; Ogden and Moradi, 2021; Chen et al., 2022). In this work, the YidC independent insertion mechanism was studied using non-equilibrium targeted MD (TMD) as implemented within the colvars module of NAMD (Fiorin et al., 2013). A TMD simulation was performed on the final conformation of the pose1 system obtained from the 550 ns equilibrium trajectory in order to transfer the Pf3 coat protein to the periplasmic side of the membrane, as seen in pose2. The RMSD collective variable was used in the TMD simulation. As a collective variable, we used the RMSD of Pf3 coat protein backbone atoms from the last frame of pose2’s equilibrium simulation trajectory. The TMD simulation was run for 100 ns with a force constant of 44 *kcal*/*mol*/Å^2^. To ensure conformational accuracy, the final frame of the targeted MD simulation was equilibrated for 20 ns without any restraints.

## 3 Results and discussion

### 3.1 YidC undergoes major conformational changes in Sec-independent insertion process

A protein must undergo various local and global conformational changes in a mechanism. A set of approximate docking models (Figure 2A) of YidC and Pf3 coat protein were developed to represent the insertion process.The docking postures created for this study were constructed based on hypotheses previously proposed in the literature (Kumazaki et al., 2014a; Chen et al., 2014; Dalbey et al., 2014; Tsukazaki, 2019), that C1 and C2 loop regions could detect the substrate initially during YidC SecY-independent insertion. Because of electrostatic and hydrophilic interactions between the substrate and YidC, the substrate is then momentarily trapped within the YidC groove. Following that, the captured substrate protein is transferred from the cavity into the membrane through hydrophobic interactions between membrane lipids and the protein. We utilized MOE docking software to produce 20 distinct docking positions that were sorted according to docking scores. For the simulations, we chose the top posture (i.e., pose1) and the eighth pose (i.e., pose2) from the 20 docked positions. The top posture was chosen since it was rated first by MOE and represented an intermediate stage of insertion. On the other hand, the 8th pose was selected since it was the highest ranked pose among the ones representing a late stage of insertion. MD simulations of these docking models were performed to investigate various conformational characteristics to see how YidC and Pf3 coat protein altered conformational properties during the insertion process. Several measures or quantities linked to local and global conformational properties were evaluated and monitored for the two conformational poses developed in this study. The C*α* root mean square deviation (RMSD) of the systems were evaluated independently of the framework to test the impact of the Pf3 coat protein on YidC’s global structure. It was found that the Pf3 coat protein had a relatively comparable RMSD in the two models simulated in this study (Figure 2D). However, the YidC protein fluctuated more in pose 1 (Figure 2C) than in pose 2 (Figure 2C). At the start of a process or mechanism, a protein is anticipated to undergo substantial conformational changes. The fact that YidC’s RMSD in pose 1 (Figure 2C) is 2 Å greater than that in pose 2 (Figure 2C) suggests that YidC goes through significant conformational changes at the start of the process. In a recent computational analysis reported by us, the RMSD of the YidC without Pf3 coat protein was determined to be less than 4 Å, which is lower than what we observed in the presence of Pf3 coat protein (Harkey et al., 2019). The lower RMSD in the absence of Pf3 coat protein supports our hypothesis that YidC protein undergoes major conformational changes in the presence of Pf3 coat protein. This demonstrates that the effect of Pf3 coat protein insertion into the membrane differs depending on the stage of the process. Although we see comparable RMSD of Pf3 coat protein in both poses, the Pf3 coat protein bending angle analysis (Figure 2A) more clearly suggests a structural difference between the two states in support of our hypothesis. The bending angle indicates that Pf3 coat protein has a lower bend at the start of the insertion process and changes its conformation inside the YidC groove (Figure 2A) as it progresses deeper into the groove. This brings us to the conclusion, that Pf3 coat protein undergoes major conformational changes to adapt to the YidC groove environment during the insertion process. In the next investigation, additional local components of YidC were rigorously investigated to elucidate more details of the insertion process.

### 3.2 Widening of the transmembrane domain is essential for incorporation of proteins in membrane during the insertion process

**FIGURE 3:**
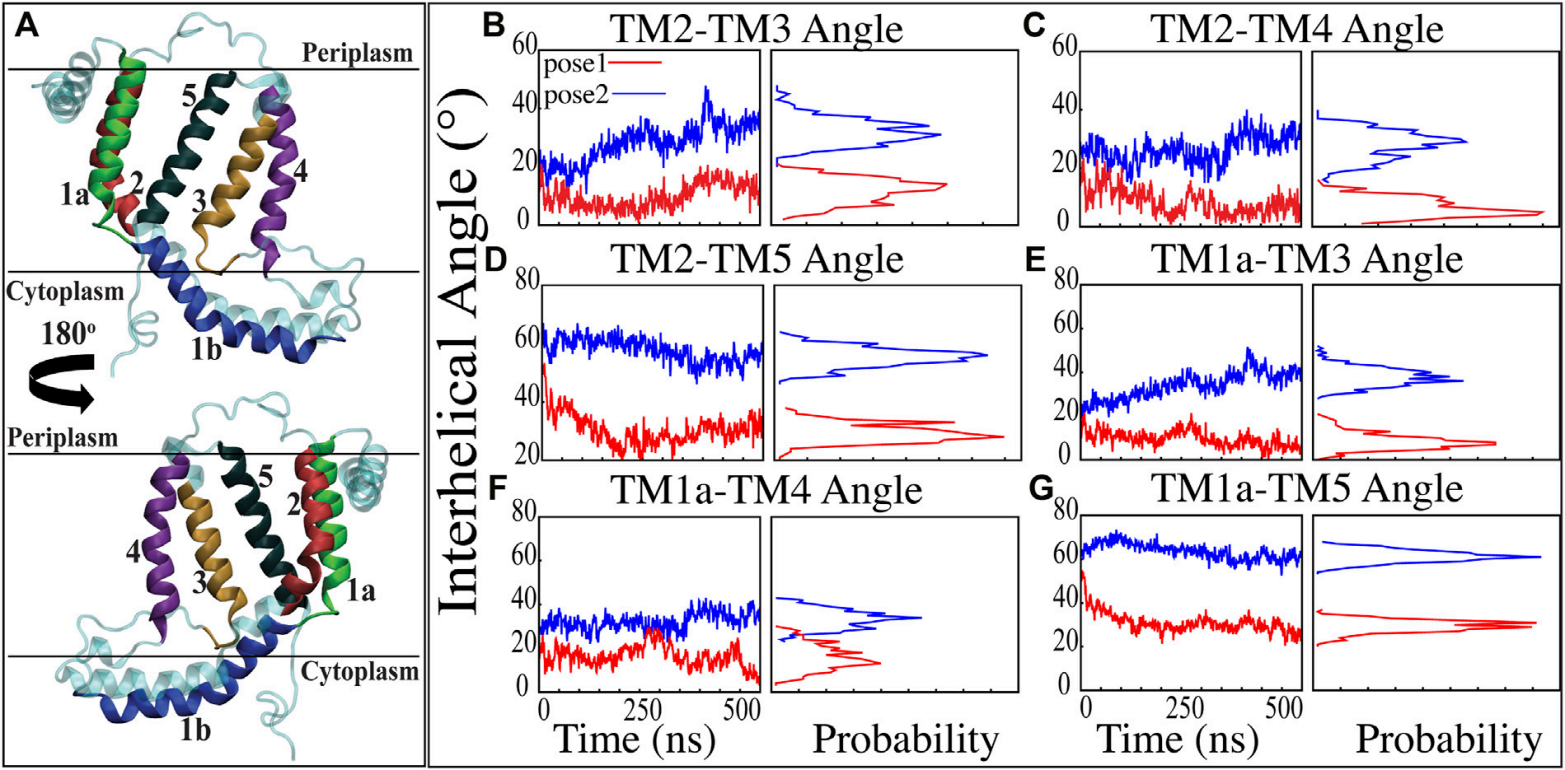
Inter-helical angles between trans-membrane helices of YidC in both docking model simulations. **(A)** Graphical representation of YidC protein on periplasmic, cytoplasmic and transmembrane regions labeled with helical numbers in the transmembrane region. **(B–D)** Overall inter-helical angle between transmembrane helix 2 and other helices of the protein in pose 1 (red) and pose 2 (blue) simulations. **(E–G)** Overall inter-helical angle between transmembrane helix 1a and other helices of the protein in pose 1 and pose 2 simulations. Also, the probability density distribution is shown for all graphs.

Previous studies have revealed that the YidC transmembrane (TM) region is crucial for membrane protein insertion into the lipid bilayer (Chen et al., 2002; Yu et al., 2008). The helical angle between each TM pair was determined in this study. In the pose2 docking simulation, the transmembrane helices (TM1a, TM2, TM3, TM4, and TM5) seem more slanted than in the pose1 docking simulation, which has a difference in the angle of over 10 degrees (Figures 3B–G). This implies that the central TM groove of YidC is substantially enlarged during the insertion of Pf3 coat protein into the membrane bilayer. The critical helices TM1a (Figures 3E,F,G) and TM2 (Figures 3B,C,D) undergo significant modifications following peptide insertion because they are stretched onto the cytoplasmic side of the membrane, which is the entrance point of Pf3 coat protein. Based on this, one may assume that throughout the insertion process, YidC experiences a gradual and tranquil conformational shift, organically adjusting to the incoming peptide. In this scenario, Yidc progressively expands its transmembrane groove to make room for the incoming peptide and then returns to a normal state once the peptide is fully incorporated into the membrane.

**FIGURE 4:**
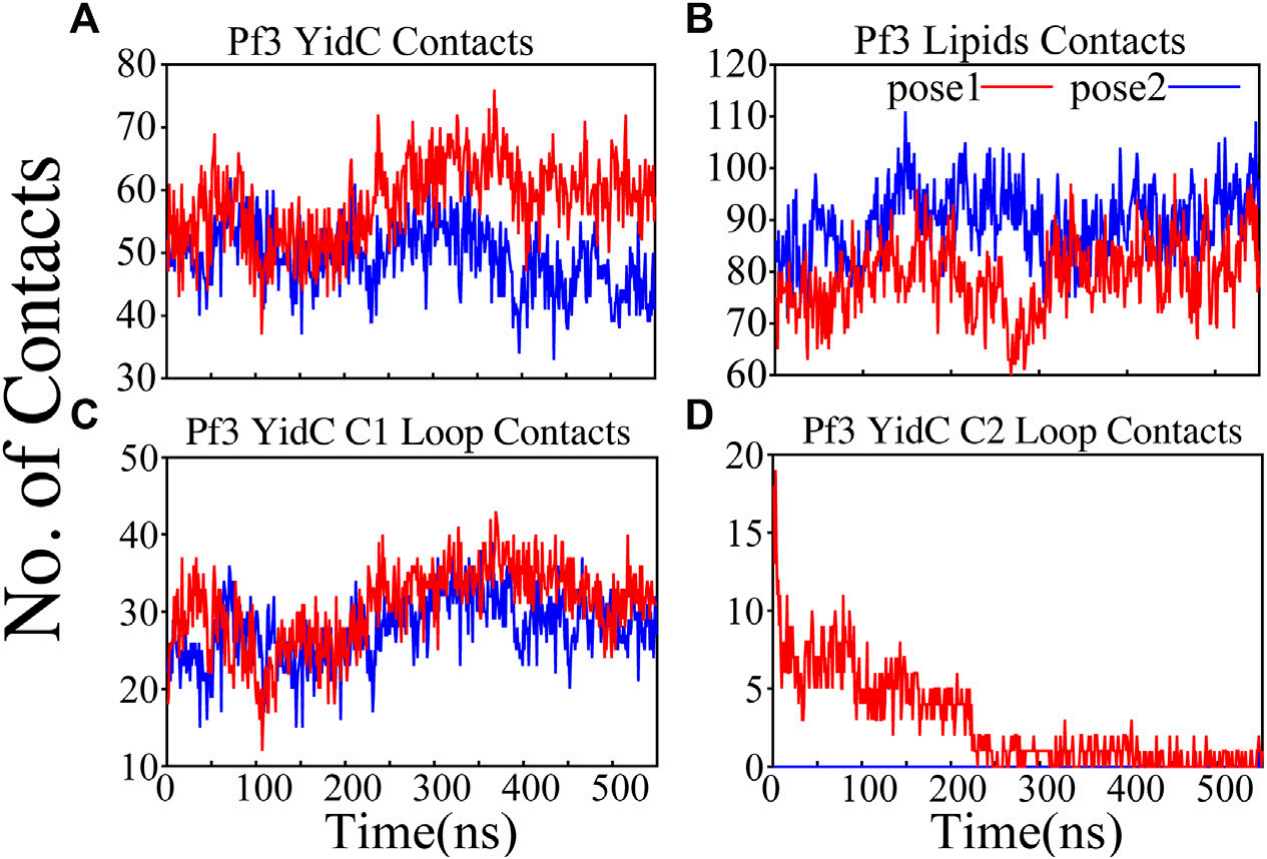
Pf3 coat protein overall interaction with YidC and POPE lipid tails in both the docking model simulations. **(A,B)** Respective number of YidC and lipid interactions with the Pf3 coat protein in pose1 (red) and pose2 (blue), shown as a function of time. **(C,D)** Number of contacts between Pf3 coat protein and the C1 and C2 loops of YidC, shown as a function of time.

The interactions of the membrane and YidC with the Pf3 coat protein were studied to learn more about the insertion process. The number of interactions of Pf3 coat protein with YidC and the membrane within 3 Å were taken into account for the interaction analysis (Figures 4A,B). The contact of the Pf3 coat protein with the membrane determines its position in the bilayer system; the Pf3 coat protein positioned inside YidC’s hydrophilic groove has a slightly higher lipid interaction distribution than the Pf3 coat protein positioned just outside the groove area (Figure 4B). Because the Pf3 coat protein is now ready to be incorporated into the membrane, Pf3 coat protein has a high degree of contact with the membrane in pose2. Previous experiments have shown that the hydrophobic interaction between the substrate and the lipid aliphatic chains can make it easier for a substrate to get inside the membrane (Gallusser and Kuhn, 1990; Kiefer and Kuhn, 2007; Kumazaki et al., 2014a; Shimokawa-Chiba et al., 2015). Here, our analysis shows that the N-terminus of the Pf3 coat protein interacts with lipids better in pose2 than in pose1 (Supplementary Figure S3A). As the Pf3 coat protein progresses through the insertion mechanism, it establishes hydrophobic interactions with aliphatic chains, and these hydrophobic interactions could aid in the insertion of the protein. The divergence of Pf3 coat protein lipid interactions supports the suggested mechanistic models for the YidC independent insertion pathway in this study. In addition to lipid interactions, the interactions between YidC and Pf3 coat protein are also important in this process. The distribution of such interactions should confirm the outcomes of the lipid interactions. Because of the greater distribution of lipid contacts in the pose2 model, Pf3 coat protein decreases its interaction with the YidC protein by shifting closer to the lipid bilayer (Figure 4B). However, at the pose1 stage of the insertion process, the association of YidC and Pf3 coat protein should be significantly strengthened before establishing the peptide in YidC’s hydrophilic groove. This would explain the YidC higher interactions with the Pf3 coat protein that were observed in the pose1 model (Figure 4A).

### 3.3 Interaction of C1 and C2 loops with Pf3 coat protein stabilizes the insertion process

Cytoplasmic loops C1 and C2 (Figure 1A) are important components in YidC’s independent insertion mechanism. Previous studies on YidC with or without the C2 loop found that its presence stabilizes YidC in the membrane (Harkey et al., 2019). In both the pose1 and pose2 models, the YidC cytoplasmic loop C1 interacts with the Pf3 coat protein. This interaction aids in the stability of the Pf3 coat protein inside YidC’s hydrophilic groove. Furthermore, these loops establish a strong interaction to retain the incoming proteins inside YidC’s U-shaped groove (Figure 4C,D). At the beginning of the insertion mechanism, the cytoplasmic C1 loop, which is deeply expanded into the cytoplasmic side, creates extremely strong contacts with the Pf3 coat protein (Figure 4C). These interactions between the C1 loop and the peptide are critical for keeping the peptide under control during the insertion process. According to our contact analysis results, the C2 loop contacts (Figure 4D) are formed only in the pose1 model, since it is the starting point of the insertion process, and a high number of protein interactions are necessary to stabilize such a long peptide. As the process progresses, the C2 loop loses its interactions (Figure 4D) with the incoming peptide once the peptide is settled inside the U-shaped groove of YidC, as seen in pose2. Thus far, we have shown that YidC undergoes significant global and local conformational changes, such as TM domain expansion, and its interactions with Pf3 coat protein, specifically through contacts in the cytoplasmic loop region. All the findings presented above confirm the major hypothesis of the study on YidC conformational changes throughout the independent insertion process.

**FIGURE 5:**
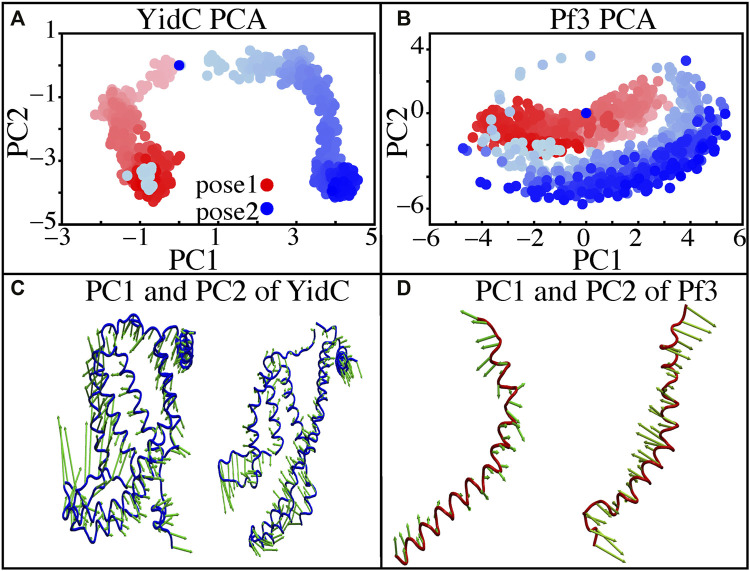
Principal component projections along PCs 1 and 2. **(A,B)** PC1 vs. PC2 for pose1 (red) and pose2 (blue) models of YidC and Pf3 coat protein. **(C)** Structural variation in PC1 and PC2 of YidC, respectively. **(D)** Structural variations in PC1 and PC2 of Pf3, respectively. The bidirectional arrow shows the direction of the fluctuation of the structure and the length of the arrow reflects the magnitude of the fluctuation. The color shading in the picture indicates a timeline, with light and dark shades representing the beginning and end of the simulation, respectively.

Principal component analysis (PCA) was used to identify the key differences between the pose1 and pose2 models. Pose1 and pose2 systems were clearly differentiated by projections onto principal components (PCs) 1 and 2. Only YidC C_*α*_ atoms are considered in this study. PC1 and PC2 contributed 49.9 and 18.8 percent of the total variance, respectively (Figure 5A). As expected, the structural analysis of pose1 and pose2 models contradicts each other in PC1 and PC2, which is logical given the significant conformational differences (Figure 5A) observed previously. The Pf3 coat protein, on the other hand, has clustered similarly along PC1 (Figure 5B). However, the fluctuation of Pf3 coat protein is different around PC2 (Figure 5B), which may be the result of a shift in Pf3 coat protein interactions and conformational changes (PC1 and PC2 contributed 45.9 and 25.4 percent of the total variance). To demonstrate this visually, square displacements of PC residues were projected onto the structure, as seen in Figures 5C,D. Overall, the major finding of the PC analysis was that the behavior of the pose1 and pose2 proteins differed considerably. This confirms our previous notion that YidC conformational dynamics play an important role in the insertion process. The PCA results are consistent with the early evidence for global and local structural changes.

### 3.4 YidC’s hydrophilic groove hydration and dehydration are critical in the insertion mechanism

**FIGURE 6:**
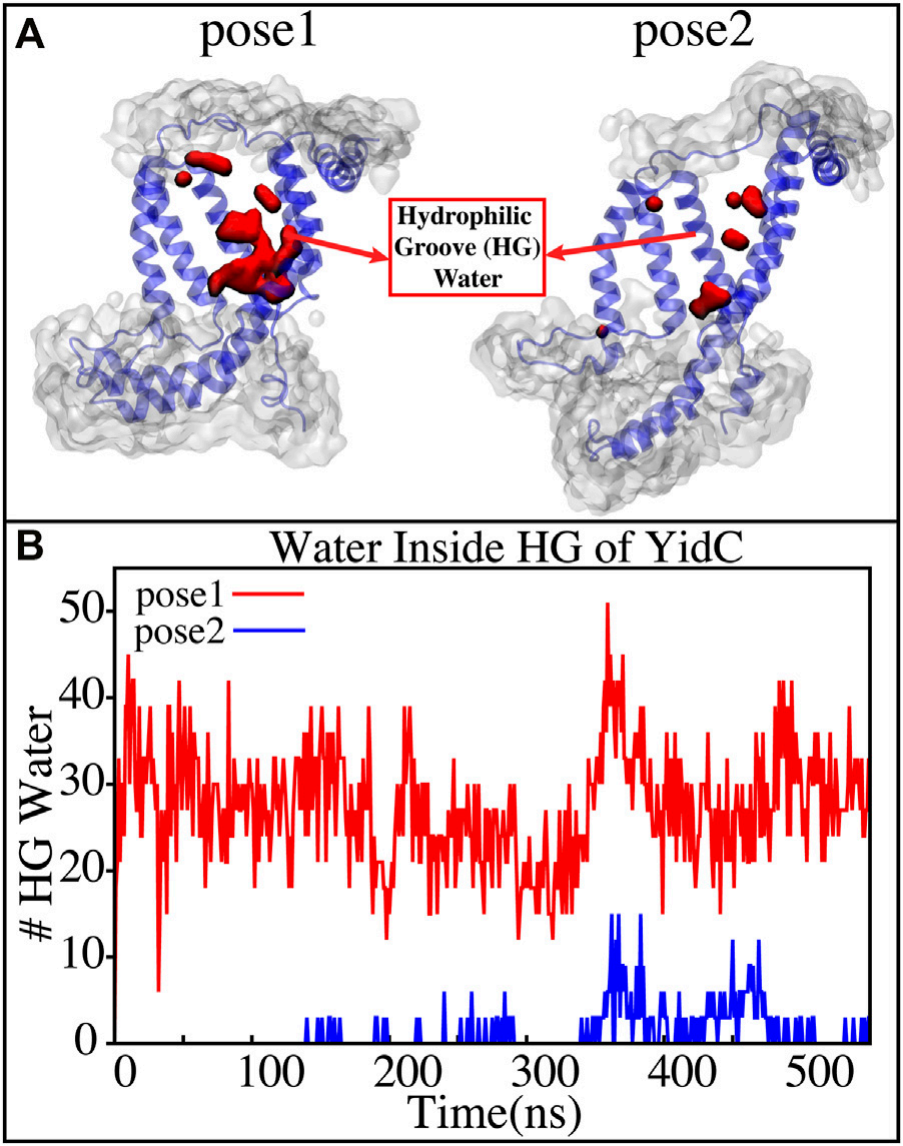
**(A)** Graphical representation of the docking models showing the average water molecule count in the hydrophilic groove (HG) region of YidC. **(B)** Number of water molecules inside the hydrophilic groove (HG) region of YidC in docking poses 1 (red) and pose 2 (blue).Pf3 coat protein entry into the TM area is aided by the water in the groove, which creates a water slide which aids in further insertion of Pf3 coat protein into the groove.

YidC has a U-shaped hydrophilic groove that is closed on the periplasmic side but exposed to the cytoplasmic side of the membrane bilayer. To examine the water content of the groove within helices TM1-TM5 (Figure 6A), the number of water molecules inside the groove region of the YidC protein was measured and plotted over the simulation time. The water analysis results reveal that the number of water molecules within the groove region is higher in pose1, which is considered the starting state of the insertion process. Whereas in the docking model pose2, the water content is close to zero throughout the simulation (Figure 6B). This confirms the previous hypothesis that a water slide motion is important in the initial positioning of the Pf3 coat protein (Wickles et al., 2014; Ito et al., 2019). The peptide enters the YidC groove via the cytoplasmic side of the membrane bilayer; the central TM helices are then widened to form a water slide (Dalbey et al., 2014; He et al., 2020; Steudle et al., 2021) and the YidC groove region is filled with water to provide a smooth sliding motion for the entering protein. As Pf3 coat protein progresses through the insertion processes, the cytoplasmic groove of YidC becomes more compact and water molecules are pushed out of the TM groove. These two factors combine to cause a hydrophobic shift in the region, making it more susceptible to membrane insertion. Previous experimental studies have reported that the hydrophilic cavity of YidC reduces the energy barrier associated with the insertion of the substrate by shortening the hydrophobic core of the membrane (Shimokawa-Chiba et al., 2015; Chen et al., 2017). Based on our results, we hypothesize that the Pf3 coat protein is initially stabilized in the groove by hydrophilic interactions (Shimokawa-Chiba et al., 2015; Chen et al., 2017), and dehydration of the groove, later in the process, will aid in breaking interactions with YidC (Figure 4A) to facilitate the translocation of Pf3 coat protein from the groove into the membrane.

### 3.5 The saltbridge interaction of Pf3 coat protein with YidC R72 in the hydrophilic groove is a significant event in the insertion process

**FIGURE 7:**
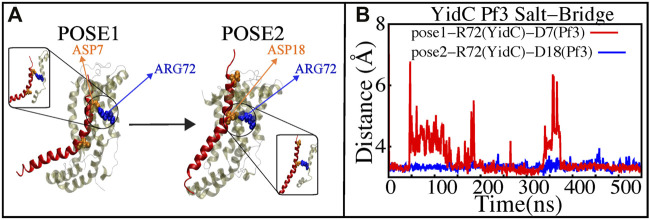
Salt—bridge connectivity of R72 (YidC) located in the groove. **(A)** Graphical representation of significant salt-bridge interactions between the Pf3 coat protein and YidC involved in the insertion process. **(B)** Distance between salt-bridge Arg72 (YidC)—Asp18 (Pf3 coat protein) and Arg72 (YidC)—Asp7(Pf3 coat protein) (labeled with blue and red lines, respectively) in YidC and Pf3 coat protein docking models.

**FIGURE 8:**
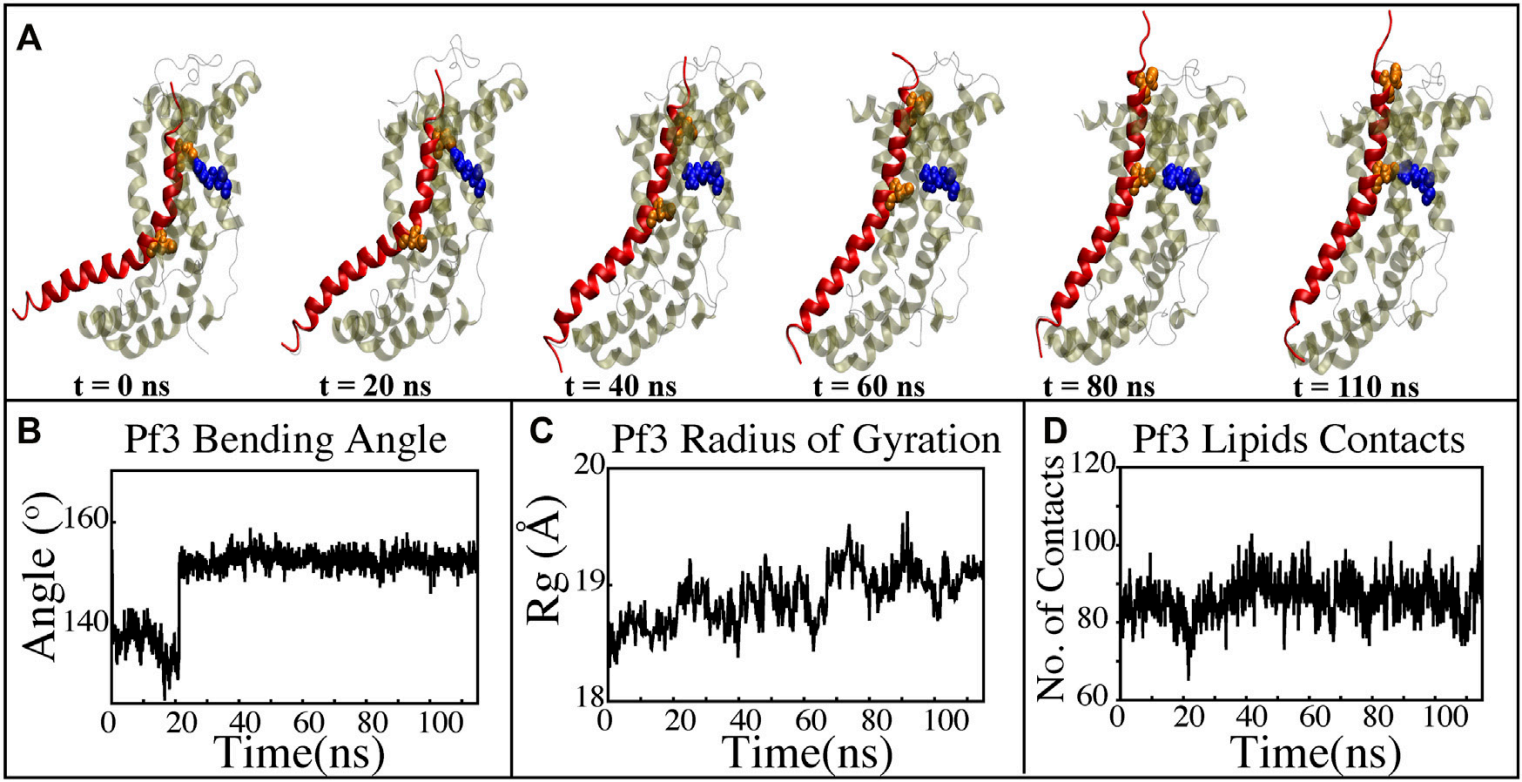
Characterizing the insertion process using targeted MD simulations. **(A)** Graphical representation of a series of targeted MD snapshots taken at different stages of the simulation. **(B)** Bending angle analysis of the Pf3 coat protein helix. **(C)** Radius of gyration of Pf3 coat protein peptide in the insertion process. **(D)** Interactions of Pf3 coat protein with lipid tails in the NE simulation process.

The YidC residue Arginine 72 (R72) is in the core cavity of the YidC transmembrane region and forms a salt-bridge with incoming protein chains. It has been suggested that before translocation, a YidC protein’s hydrophilic groove is forced into the hydrophobic cavity, implying that peptides may reach R72 for bond formation (Kumazaki et al., 2014c). According to salt bridge analysis results, R72 is available for interactions with the incoming Pf3 coat protein. During the insertion process, the R72 residue of YidC forms a stable salt-bridge with D7 and D18 of Pf3 coat protein in the pose1 and pose2 simulations, respectively (Figure 7). These two residues were experimentally shown to have an important function in the translocation of Pf3 coat protein into the membrane in an experimental research (Steudle et al., 2021). During the first phase of YidC insertion, the salt-bridge interaction between YidC’s R72 and Pf3’s D7 stabilizes the Pf3 coat proteins in the TM helical groove as soon as it enters the TM groove. As the Pf3 coat proteins move towards the periplasmic side of the protein, salt-bridge residue interactions with the Pf3 coat proteins are sequentially moved from D7 to D18 (Figure 8A).

### 3.6 Non-equilibrium simulation of YidC’s Sec-independent mechanism of Pf3 coat proteins insertion in the membrane bilayer

**FIGURE 9:**
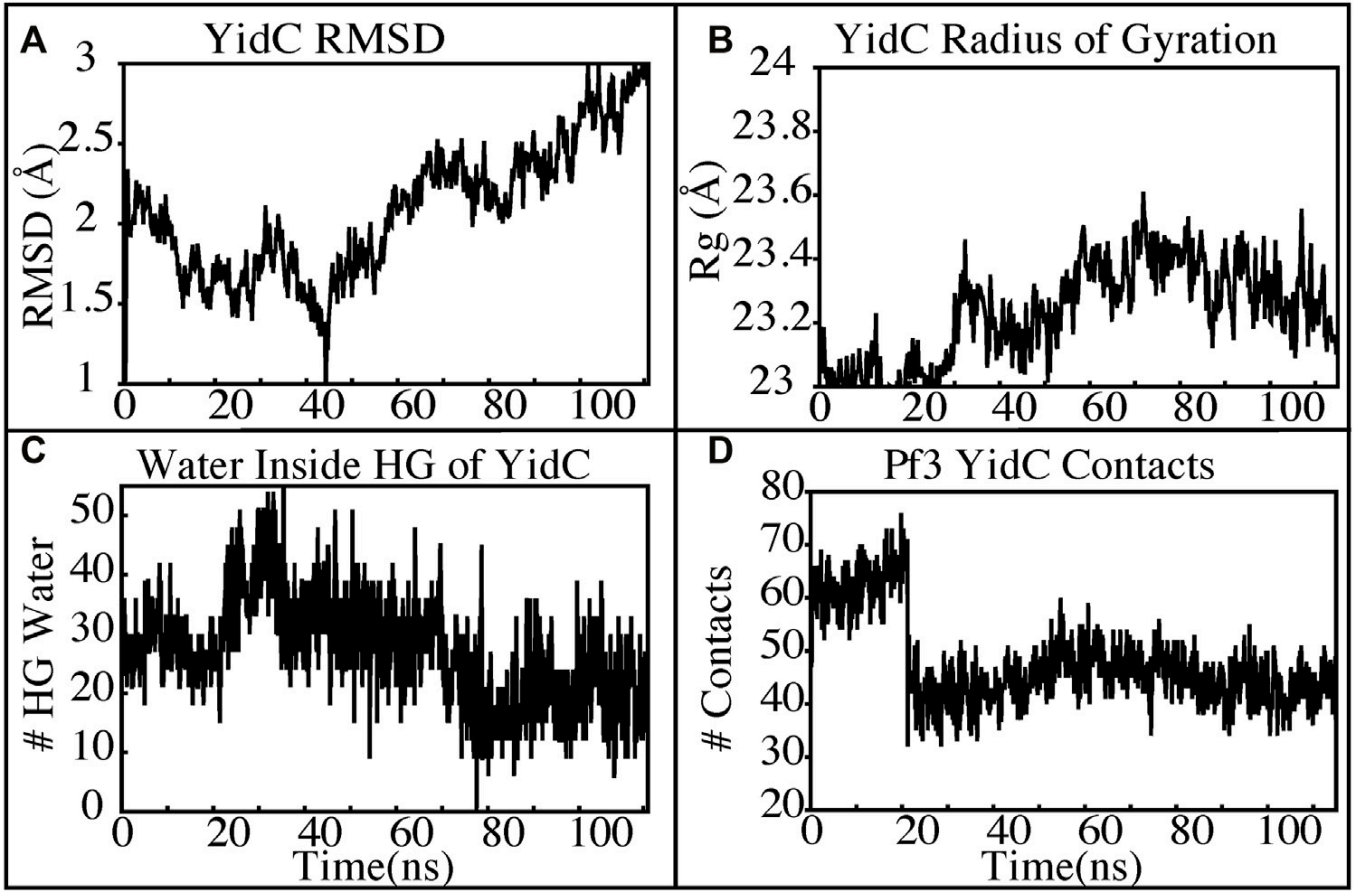
YidC conformational changes observed in the targeted MD simulations. **(A,B)** Root mean square deviation and radius of gyration of YidC in the insertion process of Pf3. **(C)** The water content in the hydrophilic groove of the protein during a 100 ns NE simulation followed by a 15 ns equilibrium simulation. **(D)** Interaction of YidC with Pf3 coat protein in the NE simulation process.

The insertion process was further investigated using the above-mentioned non-equilibrium (NE) simulation (Figure 8A) approach. Many of the key factors discussed above, such as Pf3 coat protein bending angle (Figure 8B), radius of gyration (Figure 8C), Pf3 coat protein lipid interactions (Figure 8D), the presence of water in the groove (Figure 9C), and Pf3 coat protein contacts with YidC (Figure 9D), are evaluated for the NE simulation trajectory. Our NE simulation results are totally in agreement with results produced in equilibrium simulations. The bending of Pf3 coat protein is observed in the NE simulations, where Pf3 coat protein has gone from a lower to a greater bending angle (Figure 8B) to adapt to the groove environment. The radius of gyration analysis also confirms our hypothesis about Pf3 coat protein conformational changes during the insertion process (Figure 8C). The increase and decrease in the amount of water inside the groove significantly supports the hydration and dehydration hypothesis (Figure 9C). Robust interactions of Pf3 coat protein with lipid tails (Figure 8D) play a significant role in the insertion process. As previously stated, YidC loses connections with the Pf3 coat protein as the insertion process progresses, as seen in the NE simulations, where the number of YidC-Pf3 contacts decreases during the targeted MD simulation (Figure 9D). During the insertion of Pf3 coat protein inside the membrane, YidC undergoes significant conformational changes, which we observed previously in our analysis. As expected, Yidc underwent substantial conformational changes from the beginning to the completion of the insertion process as indicated by the overall RMSD (Figure 9A) and radius of gyration (Figure 9B) analyses. Overall, based on equilibrium and NE simulations, the following mechanism for YidC’s Sec-independent insertion mechanism is proposed in this study: the incoming Pf3 coat protein first interacts with the cytoplasmic loops and gradually moves into the hydrophilic groove located in the transmembrane region, forming a salt bridge with R72. The Pf3 coat protein’s negatively charged D7 residue forms a salt bridge with the positively charged R72, which is critical to the insertion mechanism. The hydrophilic interactions within the groove (Figure 4A) and salt-bridge interactions between the negatively charged D18 residue of Pf3 coat protein and positively charged R72 of YidC would drive the Pf3 coat protein to break the initial salt-bridge and move further into the groove. The N-terminal then moves into the deep groove and dehydration of the groove takes place. The Pf3 coat protein then migrates towards the periplasmic side of the membrane, assisted by the hydrophobic force, i.e., the hydrophobic interactions of the hydrophobic regions of the Pf3 coat protein with lipid tails out of the YidC hydrophilic groove.

It is important to note that in this study, we did not attempt to investigate the entire insertion process from the initial stages of the binding to the full dissociation of the Pf3 coat protein. Instead, we only focused to look at a crucial stage of the process where the bound substrate moves up within the YidC-membrane environment. We particularly looked at the protein conformational dynamics within this part of the process. To simplify, particularly since we did not intend to investigate initial stages of binding that is likely to involve lipid headgroups, we employed a homogeneous POPE membrane instead of the anionic [phosphatidyl-glycerol (POPG) and cardiolipin (CL)] lipid-rich bacterial inner membrane. YidC’s behavior in a pure POPE membrane and a heterogeneous POPE/POPG/CL membrane was compared in a recent MD study in its *apo* state (Harkey et al., 2019). The YidC conformation and protein/lipid interactions have been shown to be unaffected by the presence or absence of the anionic lipids POPG and CL in MD simulations (Harkey et al., 2019). However, it is highly likely for the anionic lipids to play a crucial role in the initial stages of binding and insertion process (Gallusser and Kuhn, 1990; Kiefer and Kuhn, 2007; Kumazaki et al., 2014a; Dalbey et al., 2014; Shimokawa-Chiba et al., 2015; Steudle et al., 2021). Further in-depth computational and experimental studies are needed to have a better grasp on lipid specificity in the insertion process. More specifically, the proton motive force of the membrane, which is not the focus of our current study, may aid protein insertion in a lipid-specific manner. The proton motive force facilitates the YidC mediated membrane insertion by electrostatically attracting the negatively charged extracellular residues of the single-spanning membrane protein from the YidC groove, in addition to the hydrophobic interaction of the lipid tails with Pf3 coat protein (Samuelson et al., 2001; Chen et al., 2002; Kiefer and Kuhn, 2007; Kumazaki et al., 2014a; Dalbey et al., 2014; Steudle et al., 2021). The functional group attached to the phosphate moiety determines the charge of the phospholipid. Compared to zwitterionic POPE, anionic POPG and CL have a greater likelihood of protons binding to their negatively charged headgroups (Yoshinaga et al., 2016). It is likely that Pf3 coat protein in a lipid bilayer containing POPG and CL will experience stronger electrostatic attractions with enhanced proton binding compared to a pure POPE bilayer. However, the current study does not focus on the lipid-specific behavior of YidC-mediated membrane insertion, nor does it focus on the direction of the insertion, which is influenced by the proton motive force. Here we have only focused on a specific part of the insertion process, which is less dependent on proton motive force and lipid specificity and more dependent on YidC-Pf3 interactions coupled with conformational dynamics of YidC.

## 4 Conclusion

Based on our equilibrium and non-equilibrium MD simulation results, YidC must undergo major conformational changes during the SecY-independent insertion process. The incoming Pf3 coat protein would first come into contact with the cytoplasmic loops and then penetrate into the hydrophilic groove, forming a salt bridge with R72. The YidC loops on the cytoplasmic side of the bilayer are critical for moving Pf3 coat protein into YidC’s hydrophilic groove. At first, these cytoplasmic loops make contact with the Pf3 coat protein. The negatively charged D7 residue of Pf3 coat protein interacts with the positively charged R72 of YidC to form a stable salt bridge. The formation of this salt bridge is crucial in the insertion process to stabilize the Pf3 coat protein in the YidC’s TM groove. The hydrophilic interactions within the groove also aid in the passage of the protein towards the periplasmic side, which is also supported by the salt bridge between D18 of Pf3 coat protein and R72 of YidC; this combination stabilizes the position of Pf3 coat protein inside the groove. Finally, when the Pf3 coat protein is completely inside the YidC’s hydrophilic groove, it will come into contact with lipid tails. The Pf3 coat protein then travels towards the periplasmic side of the membrane, helped by the proton motive force and hydrophobic interaction with the membrane. The protein then moves into the membrane through the groove.

Despite the field’s stunning advancements in recent years and the widespread use of docking techniques, there are still a few drawbacks. The fact that model quality and docking accuracy have a substantial impact on simulation results is one of these limitations. Additional studies using more docking models, including a range of substrate proteins in various conformational states, are required to fully understand the process. Results from this study would help in creating a plan both for experimental and computational scientists to study YidC SecY-independent mechanism for deeper understanding.

## Data Availability

The original contributions presented in the study are included in the article/Supplementary Material, further inquiries can be directed to the corresponding author.
