# Supplementary material for: An investigation of the YidC-mediated membrane insertion of Pf3 coat protein using molecular dynamics simulations
Source: Front Mol Biosci. 2022 Aug 15;9:954262. doi: 10.3389/fmolb.2022.954262 (PMC9421054; doi:10.3389/fmolb.2022.954262)
Supplement: Supplementary file 1 [file DataSheet1.PDF]

frontiers

Supplementary Material

1 SUPPLEMENTARY MOVIE

1.1 Movie

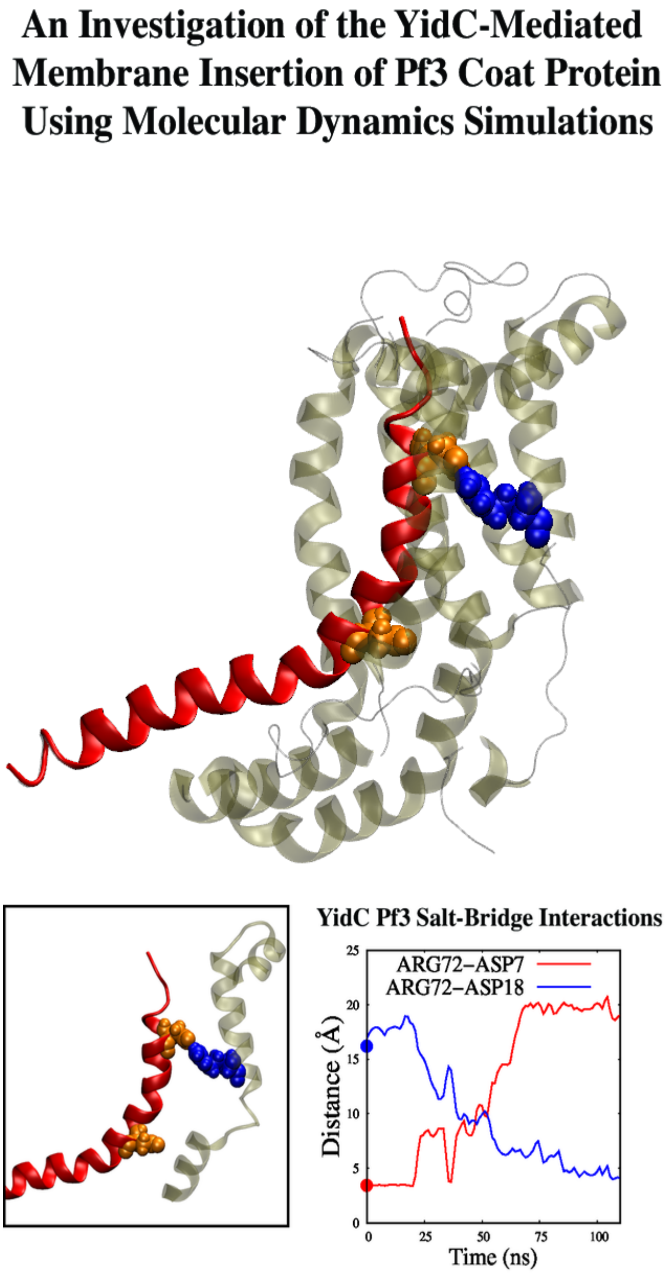

Movie. S1. Conformational transition of PF3 coat insertion process through YidC independent pathway, along with ARG72(YidC)-ASP7(PF3) salt-bridge breaking and formation of ARG72(YidC)-ASP18(PF3) salt-bridge inside the hydrophillic groove of YidC transmembrane domain. This trajectory is based on Non-equilibrium simulation.

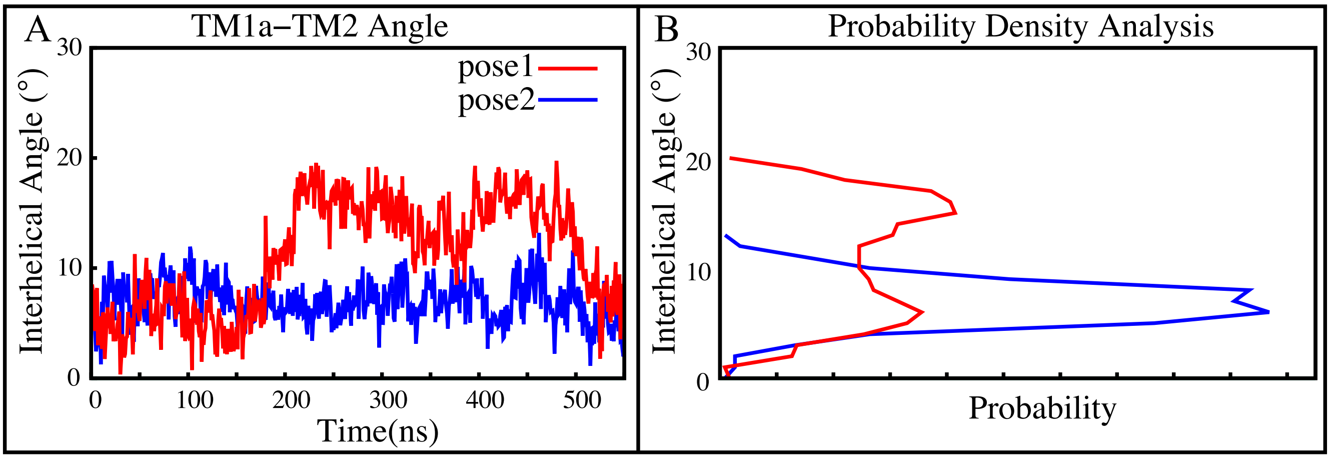

Figure. S1. (A) Overall inter-helical angle between transmembrane helix 1a and 2 helices of the protein in pose 1 (red) and pose 2 (blue) simulations. (B) The probability density distribution for the angle between transmembrane helices 1a and 2.

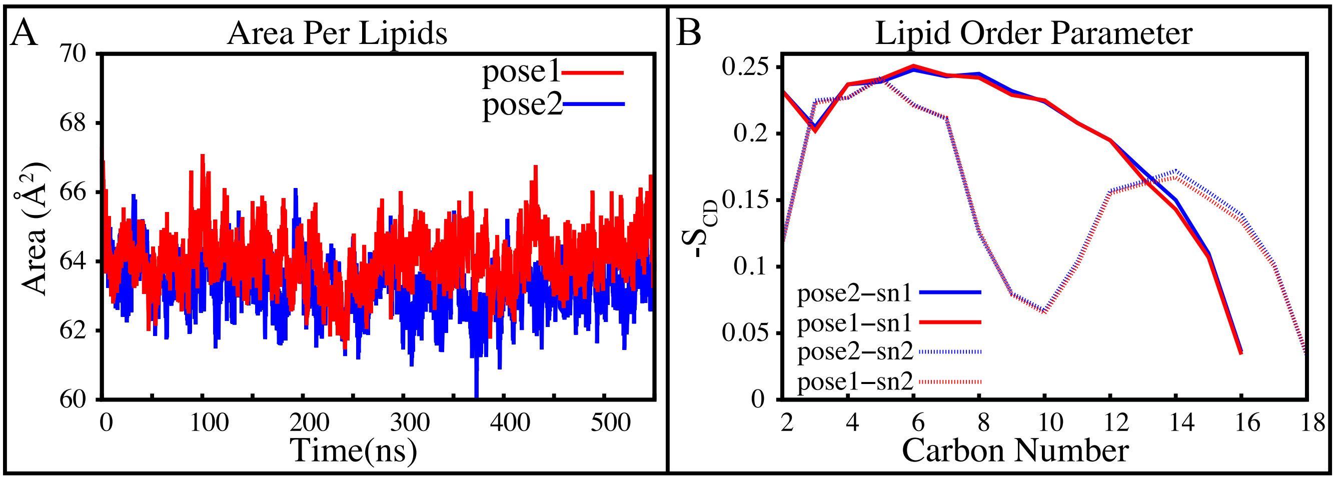

Figure. S2. (A) Time-dependent area per lipid values of the equilibrated POPE membrane in pose1 and pose2. (B) Order parameters were calculated for the equilibrated lipids POPE simulations using MEMBPLUGIN analysis tool. Results are presented for (broad line) the sn-1 and (dotted line) sn-2 chains.

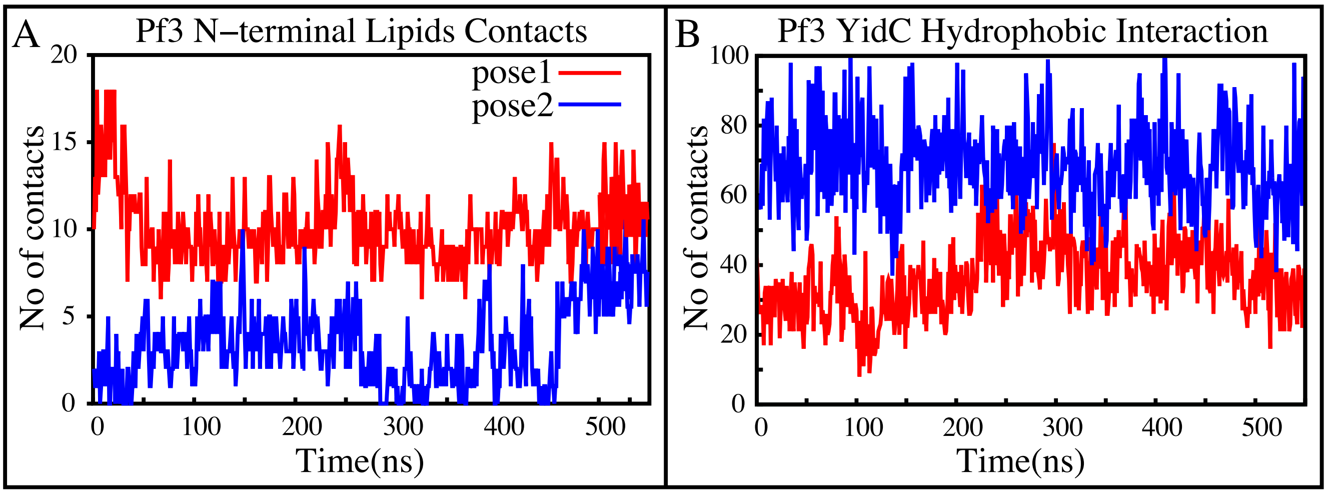

Figure. S3. (A) Pf3 coat protein N-terminal end overall interaction with POPE lipid tails in both the docking model simulations. (B) Hydrophobic residue contacts between Pf3 coat protein and YidC protein in both the docking model simulations.
